# Impact of digital health on the quality of primary care for people with chronic noncommunicable diseases: A scoping review protocol

**DOI:** 10.1371/journal.pone.0316278

**Published:** 2025-02-21

**Authors:** Pedro Bezerra Xavier, Ísis de Siqueira Silva, Renan Cabral de Figueiredo, Aguinaldo José de Araújo, Amanda Jéssica Bernardo da Silva, Severina Alice da Costa Uchôa

**Affiliations:** 1 Health Sciences Center, Postgraduate in Health Sciences, Federal University of Rio Grande do Norte, Natal, Brazil; 2 Department of Collective Health, Postgraduate Studies in Public Health, Federal University of Rio Grande do Norte, Natal, Brazil; 3 Department of Collective Health, Federal University of Rio Grande do Norte, Natal, Brazil; 4 Department of Medical Sciences, Federal University of Rio Grande do Norte, Natal, Brazil; Health Researcher, BRAZIL

## Abstract

**Background:**

Chronic Non-Communicable Diseases (NCDs) represent a significant global challenge, especially in low- and middle-income countries. The introduction of digital health in Primary Health Care (PHC) has the potential to improve the quality of care for people with NCDs by offering tools such as telemedicine, mobile applications and other information and communication technologies. The aim of this study is to identify and map global experiences of using Information and Communication Technologies (ICTs) in primary care for non-communicable diseases and assess their impact on the quality of care in PHC.

**Methods:**

This study presents a scoping review protocol based on the Joanna Briggs Institute criteria and the PRISMA-ScR guidelines. The review will be carried out in nine stages, including defining the aim and research questions, developing inclusion and exclusion criteria, and searching, selecting, extracting and analyzing the evidence. The databases consulted include MEDLINE/PubMed, JBI Evidence Synthesis and Open Science Framework.

**Results:**

The review will identify and map global experiences in the use of ICTs in primary care for NCDs and assess their impact on the quality of care in PHC. It is hoped to find studies that address the use of digital technologies for early detection, management and follow-up of NCDs, as well as their integration with traditional health systems.

**Conclusions:**

The integration of digital technologies into PHC has the potential to improve the quality of care, but it can also accentuate inequities. It is necessary to consider digital health literacy, access to tools, and legal and ethical aspects of data protection. The review will highlight the need for robust policies and adequate infrastructure to support the effective implementation of digital health in PHC.

## Introduction

Chronic Non-Communicable Diseases (NCDs), including cardiovascular, chronic respiratory, and metabolic conditions, are major contributors to global morbidity and premature mortality, disproportionately impacting low- and middle-income countries [1, 2]. Addressing these challenges requires health systems to provide efficient, cost-effective care, with Primary Health Care (PHC) playing a critical role in care coordination [3, 4].

PHC is central to reducing premature NCDs mortality, aligning with the Sustainable Development Goals to improve accessibility, community focus, and comprehensive care [5, 6]. Since COVID-19, digital health has significantly expanded within PHC, enhancing the detection, management, and monitoring of NCDs. This integration of technologies such as mobile apps and telemedicine supports personalized, equitable health services, thereby strengthening the coordination of NCDs care [7, 8].

Digital health, as defined by the World Health Organization (WHO), encompasses Information and Communication Technologies (ICTs) applications like telemedicine, mHealth, health records, Artificial Intelligence, and more [9–11]. However, while promising, technology can also deepen inequities if factors like digital literacy, easy access to the necessary tools, legal and ethical aspects in protecting the data generated and, above all, the influence of the social determinants of health on the use of this technology are not addressed [12, 13].

An exploratory search was conducted from January to April 2024 using MEDLINE/PubMed, Open Science Framework (OSF), and Google Scholar with the terms "Noncommunicable Diseases," "Digital Health," and "Primary Health Care," combined with the Boolean operator AND. This search identified eight systematic reviews on topics including: telemedicine in diabetes care for middle- and low-income countries [14]; telemedicine applications in Chronic Obstructive Pulmonary Disease (COPD) [15]; smartphone applications targeting physical activity [16]; digital tools assessing functional capacity in patients with type 2 diabetes mellitus [17]; the application of Artificial Intelligence (AI) to enhance primary healthcare [18]; health professionals’ perceptions of telehealth usability in primary care for NCDs patients [19]; quality of life analysis in asthma patients using interactive telehealth [20]; and users’ perspectives on eHealth and mHealth services in lifestyle and NCDs prevention [21].

Achieng and Ogundaini [22] conducted a scoping review on digital health and chronic disease self-management in sub-Saharan Africa, exploring region-specific implementation challenges and benefits. Additionally, Xiong et al. [23] examined the impact of factors such as political commitment, interactivity, user-centered design.

Few studies have examined the long-term effects of digital health interventions, leaving a notable gap in understanding the sustainability of these solutions and the sustained adherence of patients with NCDs [24]. Additionally, integrating digital health technologies into traditional healthcare systems and achieving interoperability across various platforms remain frequent challenges [25]. Moreover, the reliance on technology raises concerns about accessibility, particularly in regions with inconsistent internet connectivity or power supply, potentially exacerbating health disparities. Data privacy and security are also significant concerns, as the collection and storage of sensitive patient information can lead to mistrust in digital solutions if cybersecurity threats arise [26]. Furthermore, user experience plays a crucial role; poorly designed interfaces can discourage usage, leading to underutilization of these technologies and ultimately failing to achieve their intended impact [23]. Furthermore to the points already mentioned, the economic interests focused on digital health are currently concentrated in the hands of a few multinational technology corporations, mostly based in the Global North. These corporations exert enormous influence over research agendas, policies, infrastructures, and digital health technology implementations, which may limit access to the benefits of digital health for certain patient groups [26].

While existing evidence contributes significantly to evaluating digital technologies for NCDs care, studies no comprehensive scoping or systematic reviews were found that thoroughly explore the integration of digital health within national policies and systems, nor their impact on key PHC quality attributes—such as access, continuity, holistic care, management, community focus, family-centeredness, and cultural sensitivity [27], which are essential to NCDs management. Therefore, this protocol proposes a Scoping Review to identify and map digital health applications in primary care for NCDs and evaluate their impact on PHC quality.

The conceptual framework for quality will be grounded in Donabedian’s model [28], which defines health quality through three interrelated components: structure, process, and outcome. The structure encompasses the physical, human, and organizational resources for healthcare delivery, including technological infrastructure, qualified professionals, and access to devices. The process involves the activities performed by healthcare professionals, focusing on the technical, organizational, and relational dimensions of digital care. Lastly, the outcome evaluates the effects of care on patients’ and the community’s health, assessing factors such as access, effectiveness, and patient satisfaction with the care received.

This review will emphasize the necessity for robust policies and adequate infrastructure to facilitate effective and sustainable digital health implementation in PHC. It will also highlight the significance of continuous innovations to enhance NCDs management.

## Methods

This is a Scoping Review protocol based on the Joanna Briggs Institute (JBI) criteria, guided by the theoretical framework of Arksey and O’malley [29], with updates by Levac [30] and Peters [31], as well as the Preferred Reporting Items for Systematic Reviews and Meta-Analyses Extension for Scoping Reviews (PRISMA-ScR) [32, 33].

For the organization of this study, a search was conducted in the MEDLINE/PubMed, JBI Evidence Synthesis, and Open Science Framework repositories, aiming to identify knowledge gaps and previous and/or ongoing reviews that are similar. No other identical study was found. This protocol was registered in the OSF [34].

To guide the study, nine steps will be developed as recommended by JBI [31, 35]. They are: Definition and alignment of the objective and question; development of inclusion criteria with the objective and questions; description and planning of the approach for evidence search, selection, data extraction, and presentation of evidence; evidence search; evidence selection; evidence extraction; evidence analysis; presentation of results; and summarizing the evidence in relation to the review objective, drawing conclusions, and noting any implications of the results. All steps are detailed below:

### Define and align the objective and question

#### Definition of PCC

Based on the definition of the PCC mnemonic (Population, Concept, and Context) [31], available in Table 1, the objective of the research question was defined.

**Table 1:** Definition of the PCC mnemonic.

Mnemonic	Description
P^a^	People with NCDs
C^b^	Digital Health and quality of healthcare
C^c^	Primary Health Care

a–Population; b–Concept; c–Context.

#### Objective

To identify and map global experiences of using ICTs in primary care for NCDs and to assess their impact on the quality of care in PHC.

#### Research questions

- Which countries use ICTs in the care of NCDs in PHC?
- What digital resources (applications, types, and tools) are used in the care, prevention, and promotion of health for people with NCDs in the scope of PHC?
- What is the impact of digital technologies on the quality of care for people with NCDs in PHC?

#### Definition of the concepts defined in the PCC

The definition of the concepts used in this research is presented in Table 2.

**Table 2:** Description of the concepts used in this research.

Concept	Description
People diagnosed with non-communicable diseases	People diagnosed with non-communicable diseases (NCDs), which are diseases that develop over a lifetime, are generally slow, silent, and asymptomatic, but can significantly affect the quality of life and pose serious risks to individuals. The most important NCDs include cardiovascular diseases, chronic respiratory diseases (bronchitis, asthma, rhinitis), hypertension, cancer, diabetes, and metabolic diseases (obesity, diabetes, dyslipidemia) [36].
Digital Health	Digital health is a broad, economical, and secure field that applies Information and Communication Technologies (ICTs), including remote health services (telehealth) and remote consultations (telemedicine, telenursing, among others). ICTs encompasses support from computers, mobile phones, the internet, videos, text messages, websites, platforms, information systems, electronic records; mobile health (mHealth); and emerging technologies such as big data, genomics, and artificial intelligence [10].
Healthcare	Healthcare is that which attends to, respects, welcomes, and helps people who suffer—primarily due to their social vulnerability—while ensuring quality and treatment of their problems. It is an integrated work resulting from the "relationships between people," meaning it is the product of positive interactions between users, professionals, and institutions [37].
Quality of Care	The quality of healthcare is the ability of services to improve desired health outcomes for individuals and populations, based on evidence-based knowledge. To be considered high-quality, healthcare must be effective, providing evidence-based services to those who need them; safe, avoiding harm to patients; and person-centered, addressing their preferences, needs, and values [38].
Evaluation of Care Quality	The quality of healthcare will be assessed using Donabedian’s theoretical framework [28, 39] utilizing the following parameters:
	Structure: This pertains to physical resources, staff, and financial resources, as well as organizational structure, infrastructure, and normative and strategic resources (protocols, guidelines) that comprise the provision of healthcare.
	Process: These are the activities related to health care, involving the interaction between professionals and users. This component will be evaluated considering technical, organizational, and relational dimensions. Outcome: This refers to the direct changes in the health status of individuals and the community. To verify the outcomes of using digital health in primary care for people with NCDs, this study will evaluate the impact of ICTs on the essential attributes of PHC, which are first contact, longitudinality, comprehensiveness, and coordination of healthcare.

Source: Prepared by the author, 2024.

### Develop and align the inclusion criteria with the objective and questions

#### Inclusion criteria

The following will be included: a) primary studies published in full; b) grey literature; c) theses, dissertations, and official documents (governmental and from health institutions or organizations).

The search criteria will have no restrictions on time and language, which is a key characteristic of a Scoping Review. The terms and descriptors used will focus on digital health, which will impose a temporal limitation on the topic. It should be noted that this is a recent topic, and the term "digital health" has become more commonly used following the publication of the Guideline Recommendations on Digital Interventions for Health System Strengthening [10].

#### Exclusion criteria

Publications that will be excluded are duplicates, literature reviews, letters, book chapters, theoretical essays, editorials, abstracts and brief presentations, and expert opinions.

### Description and planning of the approach for evidence search, selection, data extraction, and presentation of evidence

As per JBI guidelines [31], the search methodology follows three stages: exploratory search to identify potential uncontrolled search terms, keywords, and indexed descriptors. After identifying the descriptors and keywords, an initial exploratory search was conducted in the MEDLINE/PubMed to identify publications and, after reading the titles and abstracts, identify the most commonly used descriptors and keywords (Appendix 1 in S1 File).

Following the identification of the most commonly used terms, a librarian refined the search strategy using controlled vocabularies, namely: Health Sciences Descriptors (DeCS), Medical Subject Headings (MeSH), Entree (Embase), as well as text words and subject headings, in order to obtain a comprehensive strategy and refine the results [40]. The standard search strategy is provided in Appendix 2 in S2 File.

#### Search for evidence

The standardized search strategy will be applied to search for evidence in both white and grey literature and will be adapted for each database, using the Boolean operators "AND" and "OR" as needed. The complete strategy used in MEDLINE/PubMed is available in Table 3, tested on May 04, 2024, yielding a total of 1,546 results.

**Table 3:** Search strategy used in MEDLINE/PubMed.

PCC	TERMS
**P**	"Noncommunicable Diseases"[Mh] OR "Noncommunicable Disease*"[ti] OR "Non-infectious Diseases"[tiab] OR "Non infectious Diseases"[tiab] OR "Non-communicable Disease"[tiab] OR "Non-communicable Chronic Diseases"[tiab] OR "Chronic Disease, Non-communicable"[tiab] OR "Non communicable Chronic Diseases"[tiab] OR "Non-communicable Chronic Disease"[tiab] "Diabetes Mellitus"[Mh] OR Diabetes[title] OR Hypertension[Mh] OR "High Blood Pressure*"[tiab] OR Neoplasms[MH] OR Tumor*[ti] OR Cancer*[tiab] OR "Malignant Neoplasm"[tiab] OR "Benign Neoplasm"[tiab] OR "Cardiovascular Diseases"[Mh] OR "Cardiovascular Disease*"[ti] OR "Cardiac Event*"[tiab] OR "Adverse Cardiac Event*"[ti] OR Infarction[Mh] OR Infarct*[ti] OR "Myocardial Infarction"[Mh] OR "Myocardial Infarction*"[tiab] OR "Cardiovascular Stroke*"[tiab] OR "Heart Attack*"[tiab] OR Stroke[Mh] OR Stroke*[title] OR "Cerebrovascular Accident*"[ti] OR CVA[title] OR "Cerebral Stroke"[tiab] OR "Acute Cerebrovascular Accident*"[tiab] OR "Heart Diseases"[Mh] OR "Heart Disease*"[title] OR "Cardiac Disease*"[title] OR "Heart Disorder*"[title] OR Asthma[Mh] OR Asthmas[title] OR "Bronchial Asthma"[ti] OR "Pulmonary Disease, Chronic Obstructive"[Mh] OR "Pulmonary Disease, Chronic Obstructive"[tiab] OR "Chronic Obstructive Lung Disease"[tiab] OR COAD[ti] OR COPD[ti] OR "Airflow Obstruction, Chronic"[tiab]
AND	
**C**	(Ehealth[ti] OR e-Health[ti] OR telehealth[tiab] OR Telecare[tiab] OR mHealth[ti] OR Telerehabilitation[mh] OR Telereh*[ti] OR "home telehealth"[tiab] OR "Home telecare"[tiab] OR "virtual rehabilitation*"[tiab] OR telemonitoring[tiab] OR "telecare monitoring system"[tiab] OR telenursing[ti] OR "Digital Health"[tiab] OR "Digital Health Strateg*"[tiab] OR "Digital Health Interventions"[tiab] OR "eHealth Strategies and Policies"[tiab] OR Telemedicine[Mh] OR Telemed*[ti] OR "Virtual Medicine"[tiab] OR "information and communication Technolog*"[ti])
AND	
**C**	"Primary Health care"[Mh] OR "Primary Health*"[title] OR "Health Care, Primary"[tiab] OR "Primary Healthcare"[ti] OR "Primary Care"[tiab] OR "first line care"[tiab] OR "general practi*"[ti] OR "primary medical care"[ti] OR "primary care nursing"[tiab] OR "Community mental health*"[tiab] OR "Family medicine"[ti] OR "Family physician*"[ti] OR "Community health*"[ti] OR "Community nurs*"[tiab] OR "Community pharmac*"[tiab] OR "Preventive care"[tiab] OR "Prevention program*"[tiab] OR "Preventive service*"[tiab] OR "Preventive health"[tiab] OR "Health promotion"[tiab] OR "Family health program"[tiab] OR "Family health strategy"[tiab]
TOTAL: 1,810 Results	

Source: Prepared by the author, 2024.

#### Data source

Data collection will be conducted from the following portals and databases: MEDLINE/PubMed, LILACS/virtual health library (BVS), Scopus, Web of Science, Embase, Cochrane, Epistemonikos, Google Scholar, Digital Library of Theses and Dissertations, CAPES (Coordenação de Aperfeiçoamento de Pessoal de Nível Superior) Theses Portal, DART-E (European Electronic Theses and Dissertations (ETDs)—DART-Europe), ProQuest Dissertations & Theses Global. For Google Scholar, the selection will include the first 100 files found, sorted by relevance [41]. After selecting documents from the data sources, they will be exported to the reference manager EndNote, which will be used for reference storage and removal of duplicates (documents retrieved more than once from searches in different databases).

### Selection of evidence

#### Pilot test of selection

Before the selection, a pilot test will be conducted to calibrate the reviewers, enabling greater accuracy in the selection of studies. This action will simulate the evidence selection process, where a sample of 25 articles will be independently analyzed by pairs to identify possible inconsistencies and the need for adaptation of the extraction form [31].

The extraction form (Appendix 3 in S3 File) will also be examined during the pilot test, where two team researchers will independently extract each evidence source according to the potential data items of interest, based on the PCC and the research question.

Pollock and colleagues [42] support that during the pilot test, scoping review authors should reflect on the following questions:

1. Was there anything missing from the extraction form?
2. Was there anything redundant included in the extraction form?
3. Was there anything in the extraction form that you did not understand or that could be clarified better?
4. How much time did it take you to extract the necessary information?

These points will help define the relevant data to be extracted and reach an agreement on doubts or conflicts. Thus, the instrument may be updated during the research to improve the sensitivity of evidence extraction [43].

#### Selection

The selection of studies will be based on the inclusion and exclusion criteria described above, as well as the research questions of this study. After selection, the articles used will be organized in the EndNote software, which will also identify possible duplicates [44]. The final sample of documents will be defined based on the critical reading of titles and abstracts by two independent reviewers (ISS and PBX), using the Rayyan software [45] for data organization, ensuring blinding of the reviewers, enabling the independent selection of evidence. In the event of conflicts in selection, these will be resolved by a third reviewer.

During the full-text reading stage, researchers should also pay attention to the reference lists of included studies, as these can be an additional source. If any reference is found that meets the inclusion criteria, it may be included in the sample after its reading and critical analysis.

### Extraction of evidence

#### Organization of the form

An extraction form, available in Appendix 3 in S3 File, was developed by the authors, following the guidelines of Pollock and colleagues [42], adapted to the objective and research question of this review. *Microsoft Excel®* (version 17.0) will be used at this stage to organize the extraction of information in table format. The following information will be extracted from the final sample of included evidence: Principal author; Year of publication; Country/continent/region of publication; Type of study (primary research/evidence synthesis/discussion article/official document; quantitative/qualitative/mixed methods/grey literature); Objective; Digital tool/intervention; Technology; Website; Application; or other digital tool; Purpose of use (control, screening, monitoring, reminder, consultation, etc.); Did the use of digital health impact the quality of healthcare in PHC positively/negatively?; Language of publication; Which NCDs was described?; Were any Social Determinants of Health mentioned? If so, which ones?

### Analysis of evidence

This review study will present both quantitative and qualitative results. Quantitative data will be evaluated through simple descriptive statistics (absolute frequencies and percentages) using *Microsoft Excel®* (version 17.0) [42]. The mapping results of the countries and regions identified that use digital health interventions in the care of people with NCDs will be organized and presented on a world map, showing the percentage of publications from each country/region/context, using the *MapChart software*.

The qualitative data analysis will be guided by the theoretical framework of Peters and Pollock [42, 43], who recommend using basic qualitative content analysis in scoping reviews. Open coding will be adopted to allocate concepts or characteristics into general categories [42, 43]. Scoping review studies should be guided by the three phases of qualitative content analysis [42], described by Elo and Kyngäs [46], which are: i) preparation, ii) organization, and iii) reporting.

The synthesis of evidence will be presented descriptively through tables, diagrams, and thematic maps for better visualization of the results [42]. A narrative summary will provide the mapped data and report how the results relate to the review’s objective and questions

### Presentation of results

The final report of this study will be guided by PRISMA-ScR [32], presenting the results in the form of charts, figures, or flowcharts.

#### Consultation with stakeholders

Stakeholders is the term used, in plural, to refer to the interested parties in the development of health research. It is becoming increasingly common as researchers, journal editors, and funders recognize the potential impact they have on the evidence produced [47].

During this stage, we will seek to share the preliminary results obtained, allowing stakeholders to understand and evaluate the uses and types of digital health tools used in the primary care of people with NCDs. Additionally, consulting with stakeholders will enable the authors to identify the best ways to disseminate the results and discuss how the study can help in the development of public health care policies [47]. In this way, we aim to encourage knowledge exchange, providing an opportunity to discuss new evidence or areas of research that may not have been addressed [30, 33]. In this research protocol, the sample of stakeholders will be intentionally selected using the snowball method, with 8 (eight) stakeholders: researcher (2), healthcare professional (2), technology developer (2), and people with NCDs (2), all with experience in the use of digital health aimed at primary care for people with NCDs.

#### Presentation of results: Summary of evidence in relation to the review objective, drawing conclusions, and noting any implications of the results

At this stage, a summary of the results related to the study objectives will be organized. Additionally, potential gaps will be identified based on the study results and stakeholder feedback, aiming to guide future studies on the topic. All the results and findings of the research will be presented in the scoping review that will be developed on the basis of this protocol. The results will be shared through a scientific article published in open access, and a summary of the main findings will be provided in dashboard format.

### Ethical aspects

Although the scoping review does not require ethical approval, all ethical aspects for data analysis, stakeholder consultation, including the anonymity of the information shared, and data dissemination, will be duly evidenced and respected.

## Results

**Fig 1:**
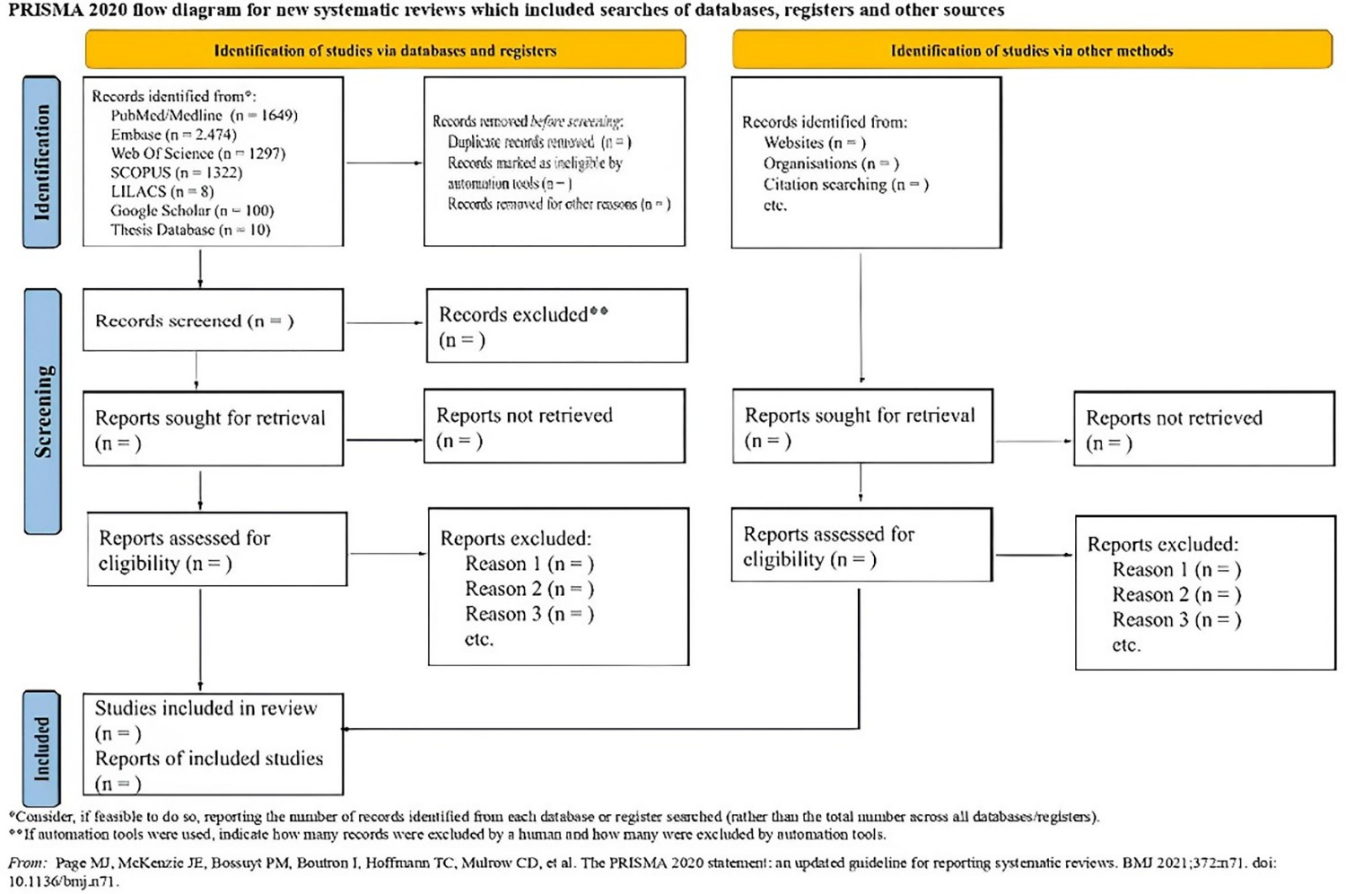
Sample selection flowchart. Source: Prepared by the authors, 2024.

The preparation of this protocol resulted in a complex and systematic search strategy, as recommended by PRISMA-ScR. The sample selection flow (Fig 1) shows the results obtained from different data sources, based on the search strategy proposed in this protocol. The complete flowchart containing all the stages of evidence synthesis will be available in the scoping review.

## Discussion

### Principal findings

The development of this scoping review protocol contributes to defining the objective and research questions. By previously defining the inclusion and exclusion criteria, search methods and analysis strategy, the protocol minimizes the risk of bias in the selection of studies and the interpretation of results [48, 49].

Technological and methodological advances in the management of NCDs indicate a significant shift towards personalized medicine and the integration of care [50]. Studies demonstrate the positive impact of digital technologies on patient education and the promotion of self-care, resulting in greater patient empowerment and better health management. This approach, however, needs to be implemented carefully so as not to alienate patients with low access to technological devices [51].

Personalization of treatment is another key part of the findings, which argue that individualized management can lead to better outcomes. This suggests that healthcare professionals need to be willing to adapt their treatment plans according to the specific needs of each patient, a challenge that requires advanced clinical skills and adequate resources [50]. In addition, non-pharmacological interventions, such as physical exercise, should be considered essential components in the treatment strategy, as they can reduce dependence on medication and improve health outcomes [52].

Although the findings are promising, there are significant limitations that need to be addressed. Most of the observed studies are focused on specific populations, limiting the generalizability of the findings to other demographic groups [53]. In addition, many studies have not fully assessed the challenges of implementing these strategies in resource-limited settings, such as rural areas or developing countries. However, the results provide a solid basis for the development of future studies that can explore personalized and integrated interventions more broadly [54].

Thus, the construction of this manuscript is based on the need to guide a serious scoping review. This protocol strengthens the organization of the review process, transparency and replicability, defining clear steps and deadlines for compliance. This facilitates the management of time and resources, increasing the efficiency of the research team. In addition, scoping reviews with well-designed protocols contribute to the identification and synthesis of a wide range of evidence, providing a comprehensive view of a topic and identifying gaps in the existing literature [35, 49, 55].

### Limitations

There are challenges typical of scoping reviews, such as the heterogeneity of the studies and difficulties in defining quality criteria. To minimize these, the inclusion and exclusion criteria have been rigorously defined. In addition, software will be used to identify duplicate texts, and a team of 3 researchers, specialized in the methodology and theme, will conduct the selection and analysis of the studies. Involving stakeholders in the presentation of results can be a challenge, as they may be biased in their interpretation of the results, placing more emphasis on one finding than another. To overcome this challenge, stakeholders will have access to the objective of the study, its methodology and its main findings, and guiding questions will guide the stakeholders’ assessment, preventing them from straying from the theme.

Difficulties related to the object of study, digital health, arising from the variability in definition in the literature and capturing the complex interactions between technologies, health professionals and patients, were also addressed. To this end, a clear concept of digital health drawn up by the WHO was adopted, guiding and delimiting the themes of the studies to be selected. As a possible gap and possibility for future studies, it would be relevant to develop primary research that seeks to explore the use of digital health in different cultural contexts, applicability, health practices and community health realities around the world. Considering that the model linking structure, process and results makes it easier to identify the interface of technologies and analyze their impact on health. Recognizing that new studies may be published during the collection period, it is proposed that the searches be updated during the data extraction and analysis stage.

### Strengths

This protocol stands out for its solid theoretical basis related to quality applied to digital health and for its methodology that follows scientifically validated guidelines. Consultation with stakeholders is a crucial point in guaranteeing the relevance, applicability and impact of the results. In this way, it helps to identify research priorities, understand the results in health management practice and indicate ways to effectively implement evidence-based interventions. The findings of the scoping review will present a comprehensive mapping of how ICTs can improve the quality of life of patients with NCDs, offering continuous and personalized support; identifying strategies to increase autonomy and self-management of chronic diseases by patients.

## Conclusion

In summary, this scoping review protocol represents a significant effort to investigate the impact of digital technologies on the management of NCDs in PHC. This work presents a robust theoretical basis and rigorous methodology aligned with scientific guidelines, and is useful for conducting a review that explores and synthesizes a diverse corpus of evidence.

The strategic inclusion of stakeholders will reinforce the relevance and applicability of the results, guiding health policies and clinical practices based on solid evidence. We recognize the inherent challenges, such as the heterogeneity of the studies and the issues in defining quality criteria, and we are committed to addressing them with methodological rigor.

## Supporting information

S1 File Appendix 1. (PDF)

S2 File Appendix 2. (PDF)

S3 File Appendix 3. (PDF)

S4 File Checklist_PRISMA-P. (PDF)

## Abbreviations

AI: Artificial Intelligence
BVS: Virtual health library
CAPES: Coordenação de Aperfeiçoamento de Pessoal de Nível Superior
DeCS: Health Sciences Descriptors
ETD: Electronic Theses and Dissertations
ICTs: Information and Communication Technologies
JBI: Joanna Briggs Institute
MeSH: Medical Subject Headings
NCDs: Chronic Non-Communicable Diseases
OSF: Open Science Framework
PCC: Population, Concept, and Context
PHC: Primary Health Care
PRISMA-ScR: Preferred Reporting Items for Systematic Reviews and Meta-Analyses Extension for Scoping Reviews
SDS: Sustainable Development Goals
WHO: World Health Organization

## References

[1] World Health Organization (WHO). Invisible numbers: the true extent of noncommunicable diseases and what to do about them. Geneva: WHO; 2022. ISBN: 9789240057661

[2] Rodrigues DLG, Belber GS, Borysow ID, Maeyama MA, Pinho APNM. Description of e-Health Initiatives to Reduce Chronic Non-Communicable Disease Burden on Brazilian Health System. Int J Environ Res Public Health 2021;18(19):10218. 10.3390/ijerph181910218 34639518 PMC8508239

[3] Briggs AM, Persaud JG, Deverell ML, Bunzli S, Tampin B, Sumi Y, et al. Integrated prevention and management of non-communicable diseases, including musculoskeletal health: a systematic policy analysis among OECD countries. BMJ Glob Health 2019;4(5):e001806. doi: 10.1136/bmjgh-2019-001806 31565419 PMC6747900

[4] Mendes EV. As redes de atenção à saúde. 2. edição. Brasília DF: Organização Pan-Americana da Saúde; 2011. ISBN: 9788579670756

[5] Organização das Nações Unidas (ONU). Objetivos de Desenvolvimento Sustentável [Internet]. Brasília, DF: ONU–Brasil; [2017]. Available from: https://brasil.un.org/pt-br/sdgs#:~:text=S%C3%A3o%2017%20objetivos%20ambiciosos%20e,no%20Brasil%20e%20no%20mundo. [accessed 2024-03–23].

[6] Organização Pan-Americana da Saúde (OPAS). Atenção Primária à Saúde [Internet]. Brasília, DF: OPAS; [202–]. Available from: https://www.paho.org/pt/topicos/atencao-primaria-saude. [accessed 2024-03-23].

[7] Silva CRDV Lopes RH, Bay OG Jr, Martiniano CS, Fuentealba-Torres M Arcêncio RA, et al. Digital health opportunities to improve primary health care in the context of COVID-19: scoping review. JMIR Hum Factors 2022;9(2):e35380. doi: 10.2196/35380 . 35319466 PMC9159467

[8] Lapão LV, Peyroteo M, Maia M, Seixas J, Gregório J, Mira da Silva M, et al. Implementation of digital monitoring services during the COVID-19 pandemic for patients with chronic diseases: design science approach. J Med Internet Res 2021; 23(8):e24181. doi: 10.2196/24181 . 34313591 PMC8396539

[9] World Health Organization (WHO). The ongoing journey to commitment and transformation: digital health in the WHO European Region, 2023. Copenhagen: WHO Regional Office for Europe; 2023. ISBN: 9789289060226

[10] World Health Organization (WHO). WHO guideline: recommendations on digital interventions for health system strengthening. Executive summary. Geneva: WHO; 2019. 31162915

[11] Silva CRDV, Lopes RH, Martiniano CS, Silva IS Uchôa SAC. Conceito de saúde digital na atenção primária à saúde (2020–2022): um estudo baseado no método evolucionário de rodgers. Bol Conjuntura 2024;17(49):432–54. 10.5281/zenodo.10565467

[12] Mumtaz H, Riaz MH, Wajid H, Saqib M, Zeeshan MH, Khan SE, et al. Current challenges and potential solutions to the use of digital health technologies in evidence generation: a narrative review. Front Digit Health Sep 2023;5:e1203945. doi: 10.3389/fdgth.2023.1203945 37840685 PMC10568450

[13] Araújo AJ, Silva IS, Figueirêdo RC, Lopes RH, Silva CRDV, Bay Junior OG, et al. Alignment and specifics of Brazilian health agencies in relation to the international premises for the implementation of digital health in primary health care: a rhetorical analysis. Front Sociol Feb 2024;9:1–7. 10.3389/fsoc.2024.1303295 PMC10881658 38390288

[14] Correia JC, Meraj H, Teoh SH, Waqas A, Ahmad M, Lapão LV, et al. Telemedicine to deliver diabetes care in low- and middle-income countries: a systematic review and meta-analysis. Bull World Health Organ Mar 2021;99(3):209–19B. doi: 10.2471/BLT.19.250068 33716343 PMC7941107

[15] Janjua S, Carter D, Threapleton C, Prigmore S, Disler RT. Telehealth interventions: remote monitoring and consultations for people with chronic obstructive pulmonary disease (COPD). Cochrane Database Syst Rev 2021;7(7)CD013196. doi: 10.1002/14651858.CD013196.pub2 34693988 PMC8543678

[16] Laranjo L, Ding D, Heleno B, Kocaballi B, Quiroz JC, Tong HL, et al. Do smartphone applications and activity trackers increase physical activity in adults? Systematic review, meta-analysis and metaregression. Br J Sports Med 2020;55(8):422–32. doi: 10.1136/bjsports-2020-102892 33355160

[17] Pepera G, Karanasiou E, Blioumpa C, Antoniou V, Kalatzis K, Lanaras L, et al. Tele-assessment of functional capacity through the six-minute walk test in patients with diabetes mellitus type 2: validity and reliability of repeated measurements. Sensors 2023;23(3):1354. doi: 10.3390/s23031354 36772396 PMC9920804

[18] Saif-Ur-Rahman KM, Islam MS, Alaboson J, Ola O, Hasan I, Islam N, et al. Artificial intelligence and digital health in improving primary health care service delivery in LMICs: A systematic review. J Evid Based Med 2023;16(3):303–20. doi: 10.1111/jebm.12547 37691394

[19] Gonçalves RL, Pagano AS, Reis ZSN, Brackstone K, Lopes TCP, Cordeiro SA, et al. Usability of telehealth systems for noncommunicable diseases in primary care from the COVID-19 pandemic onward: systematic review. J Med Internet Res 2023;25:e44209. doi: 10.2196/44209 36787223 PMC10022651

[20] Snoswell CL, Rahja M, Lalor AF. A systematic review and meta-analysis of change in health-related quality of life for interactive telehealth interventions for patients with Asthma. Value Health 2021;24(2):291–302. doi: 10.1016/j.jval.2020.09.006 33518036

[21] Bergevi J, Andermo S, Woldamanuel Y, Johansson UB, Hagströmer M, Rossen J. User perceptions of ehealth and mhealth services promoting physical activity and healthy diets: systematic review. JMIR Hum Factors 2022;9(2):e34278. doi: 10.2196/34278 35763339 PMC9277535

[22] Achieng MS, Ogundaini OO. Digital health and self-management of chronic diseases in sub-Saharan Africa: A scoping review. S Afr J Inf Manag 2022;24(1):1–8. 10.4102/sajim.v24i1.1550

[23] Xiong S, Lu H, Peoples N, Duman EK, Najarro A, Ni Z, et al. Digital health interventions for non-communicable disease management in primary health care in low-and middle-income countries. NPJ Digit Med 2023;6(1):12. doi: 10.1038/s41746-023-00764-4 36725977 PMC9889958

[24] Fragão-Marques M, Ozben T. Digital transformation and sustainability in healthcare and clinical laboratories. Clin Chem Lab Med 2023;61(4):627–33. doi: 10.1515/cclm-2022-1092 36473150

[25] Kaboré SS, Ngangue P, Soubeiga D, Barro A, Pilabré AH, Bationo N, et al. Barriers and facilitators for the sustainability of digital health interventions in low and middle-income countries: a systematic review. Front Digit Health 2022; 4:1014375. doi: 10.3389/fdgth.2022.1014375 36518563 PMC9742266

[26] Sekalala S, Chatikobo T. Colonialism in the new digital health agenda. BMJ Glob Health. 2024 Feb 27;9(2):e014131. doi: 10.1136/bmjgh-2023-014131 ; PMCID: PMC10900325. 38413105 PMC10900325

[27] Starfield B. Atenção primária: equilíbrio entre necessidades de saúde, serviços e tecnologia. Brasília: Ministério da Saúde; 2002. ISBN: 8587853724

[28] Donabedian A. The quality of care: how can it be assessed? JAMA 1988;260(12):1743–48. doi: 10.1001/jama.1988.03410120089033 3045356

[29] Arksey H O’Malley L. Scoping studies: Towards a methodological framework. Int J Soc Res Methodol 2005; 8(1): 19–32. 10.1080/1364557032000119616

[30] Levac D, Colquhoun H, O’Brien KK. Scoping studies: advancing the methodology. Implement Sci 2010;5:69. doi: 10.1186/1748-5908-5-69 20854677 PMC2954944

[31] Peters MDJ, Godfrey C, McInerney P, Munn Z, Tricco AC, Khalil H. Scoping Reviews (2020). Aromataris E, Lockwood C, Porritt K, Pilla B, Jordan Z, editors. JBI Manual for Evidence Synthesis. Adelaide: JBI; 2024. 10.46658/JBIMES-24-09

[32] Tricco AC, Lillie E, Zarin W, O’Brien KK, Colquhoun H, Levac D, et al. PRISMA Extension for Scoping Reviews (PRISMA-ScR): Checklist and Explanation. Ann Intern Med 2018;169(7):467. doi: 10.7326/M18-0850 30178033

[33] Page MJ, McKenzie JE, Bossuyt PM, Boutron I, Hoffmann TC, Mulrow CD, et al. The PRISMA 2020 statement: an updated guideline for reporting systematic reviews. BMJ 2021;372:n71. doi: 10.1136/bmj.n71 33782057 PMC8005924

[34] Xavier PB, Silva IS, Uchoa, SAC. Challenges and Opportunities of using Digital Strategies in Primary Care for Chronic Noncommunicable Diseases: a Scoping Review protocol [Internet]. Available from: osf.io/3ejdv. [accessed 2024-03-23].

[35] Peters MD, Godfrey C, McInerney P, Khalil H, Larsen P, Marnie C, et al. Best practice guidance and reporting items for the development of scoping review protocols. JBI Evid Synth 2022;20(4):953–68. doi: 10.11124/JBIES-21-00242 35102103

[36] Theme Filha MM, Souza Junior PRB, Damacena GN, Szwarcwald CL. Prevalence of chronic non-communicable diseases and association with self-rated health: National Health Survey, 2013. Rev Bras Epidemiol 2015;18(suppl 2):83–96. doi: 10.1590/1980-5497201500060008 27008605

[37] Pinheiro R, Mattos RA, organizadores. Cuidado: as fronteiras da integralidade. Rio de Janeiro: CEPESC; 2006. ISBN: 8589737241

[38] World Health Organization (WHO). Quality of Care [Internet]. Geneva: WHO; 2024. Available from: https://www.who.int/health-topics/quality-of-care#tab=tab_1. [accessed 2024-03-23].

[39] Donabedian A. The Definition of Quality and Approaches to its Assessment., Michigan: Health Administration Press; 1980.

[40] Araújo WCO. Recuperação da informação Em Saúde: construção, modelos e estratégias. ConCI 2022;3(2):100–34. doi: 10.33467/conci.v3i2.13447

[41] Godin K, Stapleton J, Kirkpatrick SI, Hanning RM, Leatherdale ST. Applying systematic review search methods to the grey literature: a case study examining guidelines for school-based breakfast programs in Canada. Syst Rev 2015; 4:138. doi: 10.1186/s13643-015-0125-0 26494010 PMC4619264

[42] Pollock D, Peters MD, Khalil H, McInerney P, Alexander L, Tricco AC, et al. Recommendations for the extraction, analysis, and presentation of results in scoping reviews. JBI Evid Synth 2023;21(3):520–32. doi: 10.11124/JBIES-22-00123 36081365

[43] Peters MD, Marnie C, Tricco AC, Pollock D, Munn Z, Alexander L, et al. Updated methodological guidance for the conduct of scoping reviews. JBI Evid Synth 2020;18(10):2119–26. doi: 10.11124/JBIES-20-00167 33038124

[44] Yamakawa EK, Kubota FI, Beuren FH, Scalvenzi L, Miguel PAC. Comparing the bibliographic management softwares: Mendeley, EndNote and Zotero. Transinformação 2014;26(2):167–76. 10.1590/0103-37862014000200006

[45] Ouzzani M, Hammady H, Fedorowicz Z, Elmagarmid A. Rayyan—a web and mobile app for systematic reviews. Sist Rev 2016;5:210. doi: 10.1186/s13643-016-0384-4 27919275 PMC5139140

[46] Elo S, Kyngäs H. The qualitative content analysis process. J Adv Nurs 2008;62(1):107–15. doi: 10.1111/j.1365-2648.2007.04569.x 18352969

[47] Concannon TW, Grant S, Welch V, Petkovic J, Selby J, Crowe S, et al. Practical guidance for involving stakeholders in health research. J Gen Intern Med 2019;34(3):458–63. doi: 10.1007/s11606-018-4738-6 30565151 PMC6420667

[48] Salvador PTCO Alves KYA, Costa TD Lopes RH, Oliveira LV Rodrigues CCFM. Contributions of scoping review in the production of the health area: reflections and perspectives. Rev Enferm Digit Cuid Prom Saúde 2021;6:1–8. doi: 10.5935/2446-5682.20210058

[49] Mattos SM, Cestari VRF, Moreira TMM. Scoping protocol review: PRISMA-ScR guide refinement. Rev Enferm UFPI 2023;12(1):3062. 10.26694/reufpi.v12i1.3062

[50] Almeida AP, Ribeiro PV, Rocha DM, Castro LC, Hermsdorff HH. Tools developed in Brazil for the promotion and assessment of adequate and healthy eating habits: A scoping review. Cienc Saude Coletiva 2023;28(11):3231–46. 10.1590/1413-812320232811.17192022 37971006

[51] Cunha LV, Silva NS, Chaves MVM, Castro TG, Bertoldi GT. Avanços Terapêuticos e estratégias de gestão para doenças crônicas não transmissíveis: uma análise das abordagens inovadoras no tratamento de diabetes, hipertensão e doenças cardíacas. Braz. J. Implantol Health Sci 2024;6(5):609–22. 10.36557/2674-8169.2024v6n5p609-622

[52] Larentis A, Barbosa D, Silva C, Barbosa J. Computação Aplicada na Assistência Educacional em Doenças Crônicas Não Transmissíveis: um Mapeamento Sistemático. In: XXX Brazilian Symposium on Computers in Education 30.; 2019; Brasília, DF: SBC; 2019. doi: 10.5753/cbie.sbie.2019.1471

[53] Malta DC, Bernal RT, Lima MG, Araújo SS, Silva MM, Freitas MI, et al. Noncommunicable diseases and the use of health services: analysis of the National Health Survey in Brazil. Rev Saude Publica 2017;51(suppl 1). doi: 10.1590/S1518-8787.2017051000090 28591353 PMC5676356

[54] Freire L, Lohanny Azevedo Viana C, Da Silva Mourão AB, Santos Abreu D, Themistocles Frazão de Araújo J. Intervenções tecnológicas usadas pela enfermagem no tratamento de doenças crônicas não transmissíveis: uma revisão integrativa. Rev Soc Cient 2024;7(1):2252–73. 10.61411/rsc202410017

[55] Silva ÍdS, Araújo AJd Lopes RH, Silva CRDV, Xavier PB, Figueirêdo RCd et al. Digital home care interventions and quality of primary care for older adults: a scoping review. BMC Geriatr 24, 507 (2024). doi: 10.1186/s12877-024-05120-z 38858634 PMC11163791

