# Supplementary material for: Impact of digital health on the quality of primary care for people with chronic noncommunicable diseases: A scoping review protocol
Source: PLoS One. 2025 Feb 21;20(2):e0316278. doi: 10.1371/journal.pone.0316278 (PMC11844851; doi:10.1371/journal.pone.0316278)
Supplement: S1 File — (PDF) [file pone.0316278.s001.pdf]

Multimedia appendix 1. Most used keywords and descriptors

Mnemonic	Synonyms/Keywords
**P**	DCNTs; Doenças Crônicas Não Transmissíveis; Chronics Diseases; Diabetes; Diabetes Mellitus; Hipertensão Arterial Sistêmica; Hipertensão Arterial; Hipertensão Sistêmica; Hipertensão; Câncer; Doenças Cardiovasculares; Infarto Agudo do Miocárdio; IAM; Acidente Vascular Encefálico; AVE; Acidente Vascular Cerebral; AVC; Doenças Cardíacas; Asma, Doença Pulmonar Obstrutiva Crônica; DPOC; COPD.
**C**	eHealth; e-Health; telehealth; Telecare; mHealth; Telerehabilitation; Telehomecare; home telehealth; Home telecare; telemonitoring; telecare monitoring system; telenursing; Digital Health; Digital Health Strategies; Digital Health Strategy; Digital Health Interventions; eHealth Strategies and Policies; Telemedicine
**C**	Primary Health Care; Primary Healthcare; Primary Care; first line care; primary care nursing; primary healthcare; primary nursing care; primary medical care; primary care.

Source: Prepared by the author, 2023.
