# Supplementary material for: Impact of digital health on the quality of primary care for people with chronic noncommunicable diseases: A scoping review protocol
Source: PLoS One. 2025 Feb 21;20(2):e0316278. doi: 10.1371/journal.pone.0316278 (PMC11844851; doi:10.1371/journal.pone.0316278)
Supplement: S2 File — (PDF) [file pone.0316278.s002.pdf]

Multimedia appendix 2. Search Strategy for Scoping Review on: Digital Resources in Health Used by Primary Care Professionals in the Prevention and Promotion of Health for Individuals with Chronic Non-Communicable Diseases

#	**Pesquisas/Searches**
**1**	"Noncommunicable Diseases"[Mh] OR "Noncommunicable Disease*"[ti] OR "Non-infectious Diseases"[tiab] OR "Non infectious Diseases"[tiab] OR "Non-communicable Disease"[tiab] OR "Non-communicable Chronic Diseases"[tiab] OR "Chronic Disease, Non-communicable"[tiab] OR "Non communicable Chronic Diseases"[tiab] OR "Non-communicable Chronic Disease"[tiab]
**2**	"Diabetes Mellitus"[Mh] OR Diabetes[title] OR Hypertension[Mh] OR "High Blood Pressure*"[tiab] OR Neoplasms[MH] OR Tumor*[ti] OR Cancer*[tiab] OR "Malignant Neoplasm"[tiab] OR "Benign Neoplasm"[tiab] OR "Cardiovascular Diseases"[Mh] OR "Cardiovascular Disease*"[ti] OR "Cardiac Event*"[tiab] OR "Adverse Cardiac Event*"[ti] OR Infarction[Mh] OR Infarct*[ti] OR "Myocardial Infarction"[Mh] OR "Myocardial Infarction*"[tiab] OR "Cardiovascular Stroke*"[tiab] OR "Heart Attack*"[tiab] OR Stroke[Mh] OR Stroke*[title] OR "Cerebrovascular Accident*"[ti] OR CVA[title] OR "Cerebral Stroke"[tiab] OR "Acute Cerebrovascular Accident*"[tiab] OR "Heart Diseases"[Mh] OR "Heart Disease*"[title] OR "Cardiac Disease*"[title] OR "Heart Disorder*"[title] OR Asthma[Mh] OR Asthmas[title] OR "Bronchial Asthma"[ti] OR "Pulmonary Disease, Chronic Obstructive"[Mh] OR "Pulmonary Disease, Chronic Obstructive"[tiab] OR "Chronic Obstructive Lung Disease"[tiab] OR COAD[ti] OR COPD[ti] OR "Airflow Obstruction, Chronic"[tiab]
3	Concept A = this line contains the group of terms for people with NCDs<br>#1 OR #2
4	(Ehealth[ti] OR e-Health[ti] OR telehealth[tiab] OR Telecare[tiab] OR mHealth[ti] OR Telerehabilitation[mh] OR Telereh*[ti] OR "home telehealth"[tiab] OR "Home telecare"[tiab] OR "virtual rehabilitation*"[tiab] OR telemonitoring[tiab] OR "telecare monitoring system"[tiab] OR telenursing[ti] OR "Digital Health"[tiab] OR "Digital Health Strateg*"[tiab] OR "Digital Health Interventions"[tiab] OR "eHealth Strategies and Policies"[tiab] OR Telemedicine[Mh] OR Telemed*[ti] OR "Virtual Medicine"[tiab] OR "information and communication Technolog*"[ti])
5	Concept B = this line contains the group of terms for Digital health resources<br>#4
6	"Primary Health care"[Mh] OR "Primary Health*"[title] OR "Health Care, Primary"[tiab] OR "Primary Healthcare"[ti] OR "Primary Care"[tiab] OR "first line care"[tiab] OR "general practi*"[ti] OR "primary medical care"[ti] OR "primary care nursing"[tiab] OR "Community mental health*"[tiab] OR "Family medicine"[ti] OR "Family physician*"[ti] OR "Community health*"[ti] OR "Community nurs*"[tiab] OR "Community pharmac*"[tiab] OR "Preventive care"[tiab] OR "Prevention program*"[tiab] OR "Preventive service*"[tiab] OR "Preventive health"[tiab] OR "Health promotion"[tiab] OR "Family health program"[tiab] OR "Family health strategy"[tiab]
7	Concept C = this line contains the group of terms for APS<br>#6
8	This line combines ABC<br>#3 and #5 and #7 (1,810 results)

Source: Prepared by the author, 2024.
