# Supplementary material for: Impact of digital health on the quality of primary care for people with chronic noncommunicable diseases: A scoping review protocol
Source: PLoS One. 2025 Feb 21;20(2):e0316278. doi: 10.1371/journal.pone.0316278 (PMC11844851; doi:10.1371/journal.pone.0316278)
Supplement: S3 File — (PDF) [file pone.0316278.s003.pdf]

Multimedia appendix 3. Extraction form

**Extraction item**	**Description**
Author	Main author
Year	Year of publication
Country/region of publication	Country/continent/region of publication
Type of evidence source	Primary research / evidence synthesis / conference abstract / discussion paper / official document
Objective	Objective
Method Used	Quantitative/qualitative/mixed methods/grey literature
Digital resource used	Tool; Intervention; Technology; Website; Application; etc... that was used
Purpose of use	What it was used for (control, tracking, monitoring, reminder, consultation, etc...)
Impact of the use of digital health on the quality of health care.	Has the use of digital health had a positive/negative impact on the quality of health care in PHC?
Language	Language of publication
NCDs described	Which NCD was described?
Do the Social Determinants of Health interfere with the use of digital health?	Yes/No, why?

Source: Prepared by the authors, 2024.
