# Supplementary material for: Impact of digital health on the quality of primary care for people with chronic noncommunicable diseases: A scoping review protocol
Source: PLoS One. 2025 Feb 21;20(2):e0316278. doi: 10.1371/journal.pone.0316278 (PMC11844851; doi:10.1371/journal.pone.0316278)
Supplement: S4 File — (PDF) [file pone.0316278.s004.pdf]

PRISMA-P (Preferred Reporting Items for Systematic review and Meta-Analysis Protocols) 2015 checklist: recommended items to address in a systematic review protocol*

Section and topic	Item No	Checklist item
**ADMINISTRATIVE INFORMATION**		
Title:<br>Identification	1a	Impact of digital health on the quality of primary care for people with chronic noncommunicable diseases: a scoping review protocol
Update	1b	Not applicable
Registration	2	Registred on Open Sience Framework (OSF). Link: DOI10.17605/OSF.IO/3EJDV
Authors:<br>Contact	3a	Authors: Pedro Bezerra Xavier[1]; Ísis de Siqueira Silva[2]; Renan Cabral Figueiredo[3]; Aguinaldo José de Araújo[4]; Amanda Jéssica Bernardo da Silva[5]; Severina Alice da Costa Uchôa[6].<br>[1] Health Sciences Center, Postgraduate in Health Sciences, Federal University of Rio Grande do Norte, Natal, Brazil. E-mail: pedrobx37@gmail.com. Orcid: https://orcid.org/0000-0002-4212-1551<br>[2] Department of Dentistry, Postgraduate studies in public health, Federal University of Rio Grande do Norte, Natal, Brazil. E-mail: isis1998.siqueira.silva@gmail.com. Orcid: https://orcid.org/0000-0002-2403-2504<br>[3] Department of Dentistry, Postgraduate studies in public health, Federal University of Rio Grande do Norte, Natal, Brazil. E-mail: cabralrenan@yahoo.com.br. Orcid: https://orcid.org/0000-0001-5172-3733<br>[4] Department of Dentistry, Graduate Program in Collective Health, Federal University of Rio Grande do Norte. E-mail: aguinaldo.araujo@ufrn.br. Orcid: https://orcid.org/0000-0002-3697-1269.<br>[5] Department of Medical Sciences, Federal University of Rio Grande do Norte, Natal, Brazil. E-mail: amandajessica.bernardo@gmail.com. Orcid: https://orcid.org/0009-0004-5356-8668<br>[6] Department of Collective Health, Federal University of Rio Grande do Norte. Natal, Brazil. E-mail: alicedacostauchoa@gmail.com. Orcid: https://orcid.org/0000-0002-2531-9937
Contributions	3b	The contributions of the authors to this manuscript are as follows:<br><br>First Author: Served as the corresponding author, leading the conceptualization of the study, writing the initial draft, editing the content, and validating the sample.<br>Second Author: Contributed to the manuscript through writing, editing, and validating the sample used in the study.<br>Third Author: Assisted in the editing process, validated the sample, and made necessary adjustments to the manuscript.<br>Fourth Author: Focused on editing and making adjustments to ensure the quality of the manuscript.<br>Fifth Author: Contributed primarily through the editing of the manuscript to enhance clarity and accuracy.<br>Sixth Author: Played a crucial role in the conceptualization of the study, contributed to the writing and editing, validated the sample, and provided overall supervision of the manuscript preparation.
Amendments	4	Not applicable

Support:<br>Sources	5a	This study was financed in part by the Coordenação de Aperfeiçoamento de Pessoal de Nível Superior – Brasil (CAPES) – Finance Code 001
Sponsor<br>Role of sponsor or funder	5b<br>5c	Not applicable<br>Not applicable
**INTRODUCTION**		
Rationale	6	From this perspective, it can be seen that few studies have investigated the long-term effects of digital health interventions, leaving a significant gap in understanding the sustainability of these solutions and the continued adherence of patients with NCDs. In addition, the integration of digital health technologies with traditional healthcare systems and interoperability between different platforms are frequent challenges that can interfere with healthcare quality. This results in difficulties in establishing efficient connections between new technologies and established health practices, limiting the potential of digital interventions to improve NCD management in the long term.<br>Although the available evidence makes important contributions to the evaluation of digital technologies used in the care of people with NCDs, no studies were found that explore the integration of digital health, national health policies and systems and the perspective of the impacts of these technologies on PHC quality attributes, such as access, continuity of care, holistic approach, care management, community focus, emphasis on the family nucleus and cultural sensitivity, which are fundamental to the management and control of NCDs.<br>With this in mind, a Scoping Review protocol is proposed, the aim of which is to identify and map the use of digital health in primary care for people with NCDs and assess its impact on the quality of health care in PHC.
Objectives	7	Definition of PCC<br>Based on the definition of the PCC mnemonic (Population, Concept, and Context), available in Table 1, the objective of the research question was defined:<br>P - People with NCDs<br>C - Digital Health and quality of healthcare<br>C - Primary Health Care<br>Objective: To identify and map global experiences of using ICTs in primary care for NCDs and to assess their impact on the quality of care in PHC.<br>Research Questions:<br>- Which countries use ICTs in the care of NCDs in PHC?<br>- What digital resources (applications, types, and tools) are used in the care, prevention, and promotion of health for people with NCDs in the scope of PHC?<br>- What is the impact of digital technologies on the quality of care for people with NCDs in PHC?
**METHODS**		
Eligibility criteria	8	Develop and align the inclusion criteria with the objective and questions<br>Inclusion Criteria

		The following will be included: a) primary studies published in full; b) grey literature; c) theses, dissertations, and official documents (governmental and from health institutions or organizations).<br>The search criteria will have no restrictions on time and language, which is a key characteristic of a Scoping Review. The terms and descriptors used will focus on digital health, which will impose a temporal limitation on the topic. It should be noted that this is a recent topic, and the term "digital health" has become more commonly used following the publication of the Guideline Recommendations on Digital Interventions for Health System Strengthening.<br>Exclusion Criteria<br>Publications that will be excluded are duplicates, literature reviews, letters, book chapters, theoretical essays, editorials, abstracts and brief presentations, and expert opinions.
Information sources	9	Data source<br>Data collection will be conducted from the following portals and databases: MEDLINE/PubMed, LILACS/virtual health library (BVS), Scopus, Web of Science, Embase, Google Scholar, Digital Library of Theses and Dissertations, CAPES Theses Portal, DART-E (European Electronic Theses and Dissertations (ETDs) - DART-Europe), ProQuest Dissertations & Theses Global. For Google Scholar, the selection will include the first 100 files found, sorted by relevance. After selecting documents from the data sources, they will be exported to the reference manager EndNote, which will be used for reference storage and removal of duplicates (documents retrieved more than once from searches in different databases).<br>Selection of Evidence<br>Pilot Test of Selection<br>Before the selection, a pilot test will be conducted to calibrate the reviewers, enabling greater accuracy in the selection of studies. This action will simulate the evidence selection process, where a sample of 25 articles will be independently analyzed by pairs to identify possible inconsistencies and the need for adaptation of the extraction form (Peters Chapter 11).<br>The extraction form (Appendix 3) will also be examined during the pilot test, where two team researchers will independently extract each evidence source according to the potential data items of interest, based on the PCC and the research question.<br>Pollock and colleagues support that during the pilot test, scoping review authors should reflect on the following questions:<br>Was there anything missing from the extraction form?<br>Was there anything redundant included in the extraction form?<br>Was there anything in the extraction form that you did not understand or that could be clarified better?<br>How much time did it take you to extract the necessary information?<br>These points will help define the relevant data to be extracted and reach an agreement on doubts or conflicts. Thus, the instrument may be updated during the research to improve the sensitivity of evidence extraction.<br>Selection<br>The selection of studies will be based on the inclusion and exclusion criteria described above, as well as the research questions of this study. After selection, the articles used will be organized in the EndNote software, which will also identify possible duplicates. The final sample of documents will be defined based on the critical reading of titles and

<table>
<tr><td></td><td></td><td>abstracts by two independent reviewers (ISS and PBX), using the Rayyan software for data organization, ensuring blinding of the reviewers, enabling the independent selection of evidence. In the event of conflicts in selection, these will be resolved by a third reviewer.
During the full-text reading stage, researchers should also pay attention to the reference lists of included studies, as these can be an additional source. If any reference is found that meets the inclusion criteria, it may be included in the sample after its reading and critical analysis.</td></tr>
<tr><td>Search strategy</td><td>10</td><td>The standardized search strategy will be applied to search for evidence in both white and grey literature and will be adapted for each database, using the Boolean operators "AND" and "OR" as needed. The complete strategy used in MEDLINE/PubMed is available in Table 3, tested on May 04, 2024, yielding a total of 1,546 results.
Table 3. Search strategy used in MEDLINE/PubMed
<table>
<tr><th>PCC</th><th>TERMS</th></tr>
<tr><td>P</td><td>(“Noncommunicable Diseases”[Mh] OR “Noncommunicable Disease*”[tiab] OR “Non-infectious Diseases”[tiab] OR “Non infectious Diseases”[tiab] OR “Non-communicable Disease”[tiab] OR “Non-communicable Chronic Diseases”[tiab] OR “Chronic Disease, Non-communicable”[tiab] OR “Non communicable Chronic Diseases”[tiab] OR “Non-communicable Chronic Dis-ease”[tiab] OR “Diabetes Mellitus”[Mh] OR Diabetes[title] OR Hypertension[Mh] OR “High Blood Pressure*”[tiab] OR Neoplasms[MH] OR Tumor*[tiab] OR Cancer*[tiab] OR “Malignant Neoplasm”[tiab] OR “Benign Neoplasm”[tiab] OR “Cardiovascular Diseases”[Mh] OR “Cardio-vascular Disease*”[tiab] OR Cardiac Event*[tiab] OR “Adverse Cardiac Event*”[tiab] OR In-farction[Mh] OR Infarct*[tiab] OR “Myocardial Infarction”[Mh] OR “Myocardial Infarc-tion*”[tiab] OR “Cardiovascular Stroke*”[tiab] OR “Heart Attack*”[tiab] OR Stroke[Mh] OR Stroke*[title] OR Cerebrovascular Accident*[tiab] OR CVA[title] OR “Cerebral Stroke”[tiab] OR “Acute Cerebrovascular Accident*”[tiab] OR “Heart Disease”[Mh] OR “Heart Dis-ease*”[title] OR “Cardiac Disease*”[title] OR “Heart Disorder*”[title] OR Asthma[Mh] OR Asthmas[title] OR “Bronchial Asthma”[tiab] OR “Pulmonary Disease, Chronic Obstruc-tive”[Mh] OR “Pulmonary Disease, Chronic Obstructive”[tiab] OR “Chronic Obstructive Lung Disease”[tiab] OR COAD OR COPD OR “Airflow Obstruction, Chronic”[tiab])</td></tr>
<tr><td colspan="2">AND</td></tr>
<tr><td>C</td><td>(Ehealth[tiab] OR e-Health[tiab] OR telehealth[tiab] OR Telecare[tiab] OR mHealth[tiab] OR Telerehabilitation[MH] OR Telereh*[tiab] OR “home telehealth”[tiab] OR “Home telecare”[tiab] OR “virtual rehabilitation*”[tiab] OR telemonitoring[tiab] OR “telecare monitoring system”[tiab] telenursing[tiab] OR “Digital Health”[tiab] OR “Digital Health Strateg*”[tiab] OR “Digital Health Interventions”[tiab] OR “eHealth Strategies and Policies”[tiab] OR Telemedicine[Mh] OR Telemed*[Mh] OR “Virtual Medicine”[tiab])</td></tr>
<tr><td colspan="2">AND</td></tr>
</table></td></tr>
</table>

		**C** — (“Primary Health care”[Mh] OR “Primary Health*”[title] OR “Health Care, Primary”[tiab] OR “Primary Healthcare”[title] OR “Primary Care”[tiab] OR “first line care”[tiab] OR “general practi*”[tiab] OR “primary medical care”[tiab] OR “primary care nursing”[tiab] OR “Community mental health*”[tiab] OR “Family medicine”[tiab] OR “Family physician*”[tiab] OR “Community health*”[tiab] OR “Community nurs*”[tiab] OR “Community pharmac*”[tiab] OR “Preventive care”[tiab] OR “Prevention program*”[tiab] OR “Preventive service*”[tiab] OR “Preventive health”[tiab] OR “Health promotion”[tiab] OR “Family health program”[tiab] OR “Family health strategy”[tiab])<br>TOTAL: 1,649 Results<br>Source: Prepared by the author, 2024.
Study records:		
Data management	11a	Data collection will be conducted from the following portals and databases: MEDLINE/PubMed, LILACS/virtual health library (BVS), Scopus, Web of Science, Embase, Google Scholar, Digital Library of Theses and Dissertations, CAPES Theses Portal, DART-E (European Electronic Theses and Dissertations (ETDs) - DART-Europe), ProQuest Dissertations & Theses Global. For Google Scholar, the selection will include the first 100 files found, sorted by relevance. After selecting documents from the data sources, they will be exported to the reference manager EndNote, which will be used for reference storage and removal of duplicates (documents retrieved more than once from searches in different databases).
Selection process	11b	The selection of studies will be based on the inclusion and exclusion criteria described above, as well as the research questions of this study. After selection, the articles used will be organized in the EndNote software, which will also identify possible duplicates. The final sample of documents will be defined based on the critical reading of titles and abstracts by two independent reviewers (ISS and PBX), using the Rayyan software for data organization, ensuring blinding of the reviewers, enabling the independent selection of evidence. In the event of conflicts in selection, these will be resolved by a third reviewer.<br>During the full-text reading stage, researchers should also pay attention to the reference lists of included studies, as these can be an additional source. If any reference is found that meets the inclusion criteria, it may be included in the sample after its reading and critical analysis.
Data collection process	11c	An extraction form, available in Appendix 3, was developed by the authors, following the guidelines of Pollock and colleagues, adapted to the objective and research question of this review. Microsoft Excel® (version 17.0) will be used at this stage to organize the extraction of information in table format. The following information will be extracted from the final sample of included evidence: Principal author; Year of publication; Country/continent/region of publication; Type of study (primary research/evidence synthesis/discussion article/official document; quantitative/qualitative/mixed methods/grey literature); Objective; Digital tool/intervention; Technology; Website; Application; or other digital tool; Purpose of use (control, screening, monitoring, reminder, consultation, etc.); Did the use of digital health impact the

Data items	12	quality of healthcare in PHC positively/negatively?; Language of publication; Which NCD was described?; Were any Social Determinants of Health mentioned? If so, which ones?<br><br>This review study will present both quantitative and qualitative results. Quantitative data will be evaluated through simple descriptive statistics (absolute frequencies and percentages) using Microsoft Excel® (version 17.0). The mapping results of the countries and regions identified that use digital health interventions in the care of people with NCDs will be organized and presented on a world map, showing the percentage of publications from each country/region/context, using the MapChart software.<br>The qualitative data analysis will be guided by the theoretical framework of Peters and Pollock, who recommend using basic qualitative content analysis in scoping reviews. Open coding will be adopted to allocate concepts or characteristics into general categories. Scoping review studies should be guided by the three phases of qualitative content analysis, described by Elo and Kyngäs, which are: i) preparation, ii) organization, and iii) reporting.<br>The synthesis of evidence will be presented descriptively through tables, diagrams, and thematic maps for better visualization of the results. A narrative summary will provide the mapped data and report how the results relate to the review's objective and questions.
Outcomes and prioritization	13	At this stage, a summary of the results related to the study objectives will be organized. Additionally, potential gaps will be identified based on the study results and stakeholder feedback, aiming to guide future studies on the topic.
Risk of bias in individual studies	14	Not applicable
Data synthesis	15a<br>15b<br>15c<br>15d	Not applicable<br>Not applicable<br>Not applicable<br>Not applicable
Meta-bias(es)	16	Not applicable
Confidence in cumulative evidence	17	Not applicable

*** It is strongly recommended that this checklist be read in conjunction with the PRISMA-P Explanation and Elaboration (cite when available) for important clarification on the items. Amendments to a review protocol should be tracked and dated.** **The copyright for PRISMA-P (including checklist) is held by the PRISMA-P Group and is distributed under a Creative Commons Attribution Licence 4.0.**

From: Shamseer L, Moher D, Clarke M, Ghersi D, Liberati A, Petticrew M, Shekelle P, Stewart L, PRISMA-P Group. Preferred reporting items for systematic review and meta-analysis protocols (PRISMA-P) 2015: elaboration and explanation. BMJ. 2015 Jan 2;349(jan02 1):g7647.
